# A scoping review of survey research with gender minority adolescents and youth in low and middle-income countries

**DOI:** 10.1371/journal.pone.0279359

**Published:** 2023-01-10

**Authors:** Isabel Pike, Cara Kraus-Perrotta, Thoai D. Ngo

**Affiliations:** 1 Department of Anthropology and Sociology, Graduate Institute of International and Development Studies (IHEID), Geneva, Switzerland; 2 Social and Behavioral Science Research and GIRL Center, Population Council, One Dag Hammarskjold Plaza, New York, NY, United States of America; University of Toronto, CANADA

## Abstract

**Background:**

Survey data that categorizes gender identity in binary terms and conflates sex and gender limits knowledge around the experience of gender minority populations, whose gender identity or expression does not align with the sex they were assigned at birth. In this review, we outline the existing survey research on the experience of a gender minority demographic for whom there is particularly limited data: adolescents and youth in low and middle-income countries (LMICs).

**Methods:**

This paper is a scoping review of peer-reviewed articles, published in English, that use survey data to examine the experience of gender minority adolescents and youth in LMICs. We conducted a search on two major databases using key terms related to gender identity, adolescence and youth, and country and region. This search yielded 385 articles. Following a team-conducted review, we retained 33 articles for the final analysis.

**Results:**

Our review shows that surveys with adolescents and youth in LMICs are increasingly including questions and taking sampling approaches that allow gender minority populations to be visible in survey data. Surveys that do so are largely focused in upper middle-income countries (n = 24), rather than lower middle-income or low-income countries, with South East Asia a notable sub-region of focus (n = 15). Sexual health, mental health, and violence are key topics of interest. Most of the surveys rely on some form of network-driven sampling focused on sexual and/or gender minorities (n = 22). The studies vary in how they ask about gender identity, both in terms of question formulation and the answer categories that are offered, as well as the extent to which they describe the questions in the article text.

**Conclusions:**

This review reveals a growing body of work that provides important insights into the experiences of gender minority adolescents and youth in LMICs. More studies could integrate these approaches, but it must be done in a way that is thoughtful about cultural and political context. Given the relatively nascent nature of such research, we encourage scholars to continue providing details on methodology, including around participant recruitment and the development of gender identity questions. This information would be valuable for researchers seeking to better include gender minorities and their experiences in survey research, but who might be daunted methodologically.

## Introduction

In recent decades, scholars and policy makers have highlighted the importance of collecting sex-disaggregated data to better understand the impact of development programming as well as crises related to conflict, disease, and climate change [1–3]. These calls have been relatively successful: survey data now frequently allows for comparisons between male and female respondents. However, conducting gender analyses with such data assumes that respondents’ gender identity corresponds to the sex they were assigned at birth. As a result, transgender and other gender minority respondents, who identify as non-binary, for example, have largely remained invisible in most survey data, limiting knowledge about their experiences as well as the ability of policy makers to institute policies and programs that can improve their well-being [4, 5].

In order to make surveys more inclusive of gender minority populations, there has been a recent burst of research that aims to develop new ways to ask about gender identity in surveys [5–10]. Surveys are gradually beginning to incorporate these measures, largely in high-income contexts, including North America, Europe and some countries in Latin America [11]. There are also some transnational initiatives underway to collect gender-disaggregated survey data that can shed light on the experience of gender minority populations, such as the People Living with HIV (PLHIV) Stigma Index. So far carried out in more than 100 countries, data from this instrument has provided important insights for policy, including around the high levels of stigma that transgender PLHIV experience [4].

Despite these growing data collection efforts, one limitation remains that surveys often require respondents to be aged 18 years and above, in part due to concerns about obtaining consent [12, 13]. This relative focus on adult populations further constrains our knowledge of gender minority adolescents and youth in LMICs. And yet, understanding the experience of the transition to adulthood is vital as it is a pivotal life phase, dense with physiological and social life events [14] and one made particularly challenging by gender minority status due to social stigma and discrimination [15]. The focus on LMICs is especially warranted given that the majority of the world’s young people live in LMICs, where they face additional stressors of poverty and extreme weather events, which will become only more intense with time [16].

In this paper, we review the emerging body of survey research that is responding to the gap in knowledge on the experience of gender minority adolescents and youth in LMICs. We focus on surveys rather than qualitative methods because the categorization inherent to survey methodology poses a greater set of challenges for applying a more nuanced approach to gender identity. In our review, we cover the broad characteristics of this research, including geographic and thematic focus, and then devote particular attention to how gender identity is asked about in surveys as well as other aspects of methodology, such as respondent sampling and survey administration. Our hope is that mapping the contours of this growing sub-field will be useful in identifying avenues for future work for researchers interested in designing surveys that can shed light on the experience of gender minority adolescents and youth in LMICs.

### Surveys and visibility

The majority of large, nationally representative surveys across the world—in high- and low-income contexts alike—do not include questions that allow transgender and other gender minority populations to be visible in the resulting data [5, 10]. Instead, most surveys offer respondents the binary answer choices of male or female and researchers then use the resulting data to conduct gender analyses, comparing the outcomes of men and women, boys and girls. The issue here is that, while connected, sex and gender are distinct concepts. Sex refers to a set of anatomical and physiological traits including genitalia, chromosomes, and hormones. Individuals are typically assigned a sex category at birth, generally male or female, based only on the appearance of their external genitalia. In turn, gender refers to the behaviors, characteristics, and status that society associates with sex; it is thus a more cultural construct and an individual’s sense of self—their gender identity—may or may not align with the sex that they were assigned at birth [8]. As such, when there is only one binary-option sex or gender survey question—and the terms are often used interchangeably—it is not possible to identify gender minority respondents in the data.

In the past decade, there has been growing awareness around the lack of visibility of gender minority populations in much survey data. As a result, there is now a rapidly growing field on how best to measure gender identity in surveys and population-level surveys have increasingly begun to include questions that assess transgender or other gender minority status [5, 8, 17, 18]. From these developments, two broad approaches have emerged. The first “expanded” approach is to provide more than two answer options to a gender identity question. Respondents may be able to choose, for example, between male, female, transgender and other. Though providing the options of male and female on a question about gender identity theoretically conflates sex and gender, it has generally been found to produce more accurate data than providing the options of man and woman [5, 19].

The second approach is the “two-step method,” in which a respondent is asked their assigned sex at birth and their current gender identity in two separate questions [5]. Data from these two questions can then be used to identify transgender respondents: those with discordant responses can be identified as transgender and those with concordant responses as cisgender. For example, a respondent who selects male at birth and then female as their current gender identity could be categorized as transgender. Tests of the two-step approach in the US have found it to be more reliable than the expanded one question option, yielding a lower level of missing responses [9]. Furthermore, this approach acknowledges both the salience of sex at birth and current gender identity to life experience and outcomes [20]. However, while the transgender construct inferred from the two questions is helpful in identifying gender minority individuals that might be missed otherwise, it is important to note that it is qualitatively different from an individual choosing “transgender” on an expanded gender identity question [5]. The latter is a clearer indication of transgender identity—it is a response to an explicit question on gender identity—while the two-step method infers this identity indirectly [8].

With both the expanded and two-step approach, the options for answer categories are not fixed, but rather adaptable to the gender dynamics of a particular time and place. This is important as categories considered inclusive in one place may potentially be a cultural imposition elsewhere, obscuring existing gender minority identities, as has been argued about the category of transgender in India [21], and it might be difficult or even impossible to translate certain questions or answer options into other languages [22]. At the same time, both question formats face the challenge of determining which fixed answer categories to offer given that, in many contexts, there are a multitude of named gender minority identities. Including a write-in “other” option can allow respondents to provide their own identity categories, but might result in data challenges around small case numbers [23, 24].

Responding to the limitations of categorical approaches, some scholars have proposed that surveys should include gradational measures of femininity and masculinity alongside questions on sex at birth and current gender identity [25]. Others have pointed out that including questions on gender expression might be particularly useful in studies focused on youth, who may not yet identify with named categories, but may still be gender non-conforming in ways that shape their experience [5]. Relatedly, existing research suggests that surveys with adolescents and youth might benefit from especially clear language around gender identity questions, such as providing definitions for the various categories [26].

### Survey research on gender minority populations in LMICs

Doing research focused on transgender and gender minority populations can be additionally challenging in many LMICs. Amongst individuals working on transgender student rights globally, Jones [27] found that concerns about assuring research participants’ safety as well as about political backlash were particularly acute amongst those from the Global South. Respondents noted that public knowledge of an individual’s gender identity can endanger their employment, safety, and lives.

However, these challenges vary across LMICs with considerable differences in the extent to which gender minority populations are recognized culturally and legally. In some countries, gender minority populations, such as *travestis* in Brazil or *hijra* in India, are considered a distinct identity group whereas in other countries, trans identity tends to be more often equated with lesbian and gay identity. At times, cultural recognition maps onto greater respect for gender minority populations, but even in countries like Thailand and the Philippines, known to be relatively “transfriendly,” gender minority populations experience violence and discrimination [28, 29]. From the legal perspective, a few countries penalize the gender expression of trans people, but more often, gender minority groups are generally persecuted through other laws, including those that criminalize “cross-dressing,” homosexuality, sex work and loitering [28, 30]. The countries with the harshest laws around sexual orientation, often rooted in colonial histories, are mainly in sub-Saharan Africa, North Africa, the Middle East, and South Asia, severely impacting both sexual and gender minorities [31].

Most of the research testing new ways to ask about gender identity on surveys has focused on high income contexts. But, given the cultural specificity of gender identity, best practices developed in one setting cannot be automatically transferred to another [11]. Indeed, existing research shows that culture impacts how gender identity questions are interpreted and answered. For example, an online study on the health of men who have sex with men (MSM) in Latin America, the Caribbean, Spain, and Portugal found that respondents often equated sexual and gender identity [20]. Amongst respondents who opted for “other” on a gender identity question, the majority wrote in their sexual orientation, which the authors speculated might be a result of “gender being more tied to sexual orientation and sexual positioning in the Latin American context” [20: 1510]. The authors suggested future studies provide a definition of gender identity, differentiating it from sexual orientation, in the question prompt. However, others have pointed out that, in some countries, understandings of sexuality and gender can overlap in a way that makes it difficult to ask separate questions about sexual and gender identity [32]. Given such variation, a central aim of this review is to reveal the different ways in which scholars have studied the experience of gender minority adolescents and youth through survey research across cultural contexts.

## Methods

This paper constitutes a scoping review rather than a systematic review, given our aim to map the general contours of a relatively nascent body of research [33]. The focus is on understanding the characteristics of existing research rather than reconciling disparate results. Our unit of analysis was the article rather than the underlying survey because we were interested not only in the characteristics of the survey, but also the thematic focus of the articles as well as how the authors described and used the survey data, particularly the gender identity questions. We carried out the search on two major social and behavioral science databases (Web of Science and PubMed) in February 2022. Our search terms were in three domains: gender identity, adolescent/youth, and country/region as illustrated in Table 1.

**Table 1:** Search terms.

Domain	Search terms
Gender identity	transgender OR queer OR non-binary OR gender non-conforming OR “gender expansive” OR “gender fluid” OR “third gender” OR “gender minorit*” OR “gender divers*”
Adolescent/youth	Adolescents OR young people OR young adult* OR student* OR youth OR teen*
Region	Africa OR Asia OR Latin America OR Middle East OR Afghanistan OR Albania OR Algeria OR “American Samoa” OR Angola OR Argentina OR Armenia OR Azerbaijan OR Bangladesh OR Belarus OR Belize OR Benin OR Bhutan OR Bolivia OR “Bosnia and Herzegovina” OR Botswana OR Brazil OR Bulgaria OR “Burkina Faso” OR Burundi OR “Cabo Verde” OR Cambodia OR Cameroon OR “Central African Republic” OR Chad OR China OR Colombia OR “Comoros” OR Congo OR “Costa Rica” OR “Cote d’Ivoire” OR Cuba OR Djibouti OR Dominica OR "Dominican Republic" OR Ecuador OR Egypt OR “El Salvador” OR “Equatorial Guinea” OR Eritrea OR Eswatini OR Ethiopia OR Fiji OR Gabon OR Gambia OR Georgia OR Ghana OR Grenada OR Guatemala OR "Guinea Bissau" OR Guyana OR Haiti OR Honduras OR India OR Indonesia OR Iran OR Iraq OR Jamaica OR Jordan OR Kazakhstan OR “Kiribati” OR Kenya OR “North Korea” OR Kosovo OR “Kyrgyz Republic” OR Lao OR Lebanon OR Liberia OR Lesotho OR Libya OR Madagascar OR Malawi OR Malaysia OR Maldives OR Mali OR “Marshall Islands” OR Mauritania OR Mauritius OR Mexico OR Micronesia OR Moldova OR Mongolia OR Montenegro OR Morocco OR Mozambique OR Myanmar OR Namibia OR Nepal OR Nicaragua OR Niger OR Nigeria OR “North Macedonia” OR Pakistan OR “Papua New Guinea” OR Paraguay OR Peru OR Philippines OR Romania OR Russia OR Rwanda OR Samoa OR “Sao Tome and Principe” OR "Senegal" OR Serbia OR “Sierra Leone” OR “Solomon Islands” OR “Somalia” OR “South Africa” OR “South Sudan” OR “Sri Lanka” OR “St. Lucia” OR “St. Vincent and the Grenadines” OR Sudan OR Suriname OR Syria OR Tajikistan OR Tanzania OR Thailand OR Timor-Leste OR Tonga OR Tunisia OR Turkey OR Turkmenistan OR Tuvalu OR Uganda OR “Ukraine” OR Uzbekistan OR Vanuatu OR Venezuela OR Vietnam OR “West Bank” OR Yemen OR Zambia OR Zimbabwe

To guide the selection of articles for the review, we used the following inclusion criteria:

1. Publication language is in English
2. Quantitative or mixed methods study in a peer-reviewed journal, drawing on survey data
3. Specifically focused on gender minority adolescent or youth populations OR the study participants are not primarily adolescents or youth, but it surveys participants in-depth about their experiences during this stage of life
4. Conducted in full or in part in any low, lower-middle, or upper-middle income country, according to World Bank classification [34].

The most ambiguous of these criteria was the focus on adolescence and youth, which are distinct yet overlapping concepts. Both adolescence and youth signify the transition from childhood to adulthood but adolescence generally refers to the earlier portion of this stage of life and youth to the later years [13]. The World Health Organization (WHO), for example, defines adolescence as spanning from 10 to 19 years and youth from 15 to 24 years [35]. While it is agreed upon that these two terms encompass an important phase of transition, year-based definitions vary, particularly for the category of youth. The African Union, for example, has a wider age span than the UN, defining youth as 15 to 35 years [36]. For the purposes of this study, we included articles if the paper’s stated research objective concerned gender minority adolescent or youth, either as the primary population or a key sub-group. We included “student” as a search term in order not to miss articles that focused on adolescent or youth populations, but did not use those signifiers in the title or abstract.

As illustrated in S1 Fig, after removing duplicates, 385 articles remained. Based on a review of titles and abstracts, an additional 294 articles were removed for not meeting the inclusion criteria. Following this abstract review, the first two authors did a full-text review of the remaining 91 articles and then met to resolve any conflicting assessments. Following this step, a further 58 studies were excluded, mainly for not attending to the experience of gender minority youth (n = 46), leaving a final total of 33 articles. The 33 articles represented 29 different surveys: most papers (n = 26) were the only ones to use their survey data, but there were two cases of two papers drawing on the same survey data and one case of three papers drawing on the same survey data. To systematize our analysis, based on our reading of the articles, we created an excel spreadsheet to outline the key elements relevant to the review. In the supplementary materials, S1 Table lists the articles and their authors, title, year of publication, country, and area of focus. The table also provides details related to research design, including analytical sample size, age range, sampling strategy, survey administration, and gender identity question type, as well as indicates which articles drew on the same survey data.

## Results

The articles revealed a small but rapidly growing field—around 80 percent (n = 27) of the articles had been published since 2019 and only two articles pre-dated 2015. In this results section, we first outline some of the broad characteristics of these papers, including geographic focus and how the studies sampled respondents and administered their survey questionnaires. These elements had implications for how gender identity questions were included and discussed in the study, which is the focus of the second half of this results section. The results section concludes with a discussion of the articles’ key thematic areas of focus.

### Geographic focus

The articles tended to focus on some regions more than others. Just over 70 percent of articles (n = 24) drew on survey data from upper middle-income countries compared to around 30 percent (n = 9) from lower middle-income countries. There were no papers that that used survey data from a low-income country. The most well-represented geographic region was Asia comprising around two-thirds of the articles (n = 22). The remaining articles were from Latin America (n = 9) and sub-Saharan Africa (n = 2).

Within geographic region, certain countries stood out for having a particularly high number of studies. In the case of Asia, most of the articles focused on South East Asia (n = 15) and within this sub-region, two-thirds of the articles (n = 10) were from Thailand with the remaining portion comprised of one or two studies from Indonesia, Myanmar, the Philippines, and Vietnam. Similarly, of the studies from Latin America, six of the nine were from Brazil and three were from Mexico (one of which was a cross-national study with Chile). To some extent, this geographic distribution reflects how some of the papers used the same survey data to explore different research questions–for example, three of the 13 studies from Thailand drew on the same school-based survey.

### Sampling and administration

The majority of the studies (n = 22) employed nonrandom sampling, generally leveraging existing social networks to identify and recruit respondents. Some authors justified this approach by noting the more difficult to reach nature of transgender and LGBT populations more generally [37, 38]. Rather than survey the general population, these studies tended to focus on gender minority populations, often alongside other specific populations, such as MSM. This more targeted approach perhaps helps to explain why the sample sizes of the studies reviewed were often relatively small; just under half of the studies (n = 15) had fewer than 500 respondents.

Of the articles that relied primarily on network-based sampling (n = 22), nearly all (n = 19) discussed collaborating with institutions to recruit respondents. It was most common (n = 10) to work with local non-governmental organizations (NGOs) or community-based organizations (CBOs), which then helped through, for example, distributing online surveys and identifying seed respondents. Studies also worked with clinics (n = 3), schools and universities (n = 4) or a combination of these institutions (n = 2) to identify respondents. Two of the four articles that did not mention working with institutions at the sampling phase drew on the same survey in Brazil, which recruited participants through Facebook advertisements [39, 40].

The remaining third (n = 11) of the studies employed some form of random sampling in their research design and nearly all of these (n = 9) worked through schools or colleges to recruit their participants. For example, a study in Thailand randomly selected provinces and then secondary schools to obtain a nationally-representative study of around 32,000 secondary school students, the largest number of respondents of any study included in this review [41]. Having asked about gender identity, the data could then be used to compare mental health, drug use, sexual health, and the experience of violence between cisgender and transgender students. Another study, focused on self-harm amongst LGBT youth in Accra, Ghana, recruited the majority of respondents through schools and then incorporated out-of-school youth via charity organizations and census street enumeration areas [42].

These studies tended to have a larger number of respondents and were more likely to include cisgender heterosexual populations, allowing for comparative analysis of the experience of gender minority populations. In some cases, however, this comparative analysis was limited by the small number of transgender respondents in the final sample. For example, the Ghana study had more than 2,000 respondents but grouped transgender respondents (n = 29) with lesbian, gay, and bisexual respondents in the analysis. The authors cited the small total number of LGBT respondents (n = 74) in the sample for taking this approach [42].

In terms of survey administration, around 60 percent (n = 20) of the studies had participants complete the surveys themselves, either due to the fact that the survey was online or that participants were provided with paper or computer-assisted self-interviewing (CASI) questionnaires. Certain studies justified the self-administered approach by stating that it reduced social desirability bias and provided additional privacy [43]. The remaining studies either used in-person interviews (n = 11), a handful (n = 3) of these in combination with a self-administered approach, or did not specify how the interviews were administered (n = 2). In some cases, when surveys were conducted face-to-face, interviewers and data collectors were part of the transgender or broader LGBT community, including a survey in Myanmar on HIV testing amongst MSM [37].

### Gender categorization

Though all papers focused on the experience of gender minority youth, they varied in the extent to which they described how the surveys had captured respondents’ gender identity. Around a fifth (n = 7) provided the exact wording of the gender identity questions and the categories offered and just under a third (n = 10) did not provide the wording, but described the type of questions used and how answers were categorized. The rest (n = 16) did not discuss how gender identity was asked about in the survey questionnaire. This, in part, reflects the frequent use of respondent driven sampling and/or the limiting of the sample to certain demographics: nine studies noted gender minority identity as part of the criteria for participation in the study, often alongside MSM or LGBTQ populations more broadly. In these cases, categorization of gender identity appears to have taken place at the recruitment phase rather than as a question in the survey itself, though this is not always clear from the text. A handful of the articles (n = 3) referred the reader to other articles for further details on survey design, though without indicating whether these would include details on assessing gender identity. Explicit discussion of the questions and their options is helpful as though descriptive statistics can provide some indication of how gender identity questions were formulated, they may be misleading given that sometimes categories are grouped together at the analysis phase, as discussed above.

Of the papers that provided some detail on how gender identity was assessed, most (n = 14) used a version of the two-step approach, asking about sex at birth and current gender identity. One paper that used this approach and provided the exact wording was a paper on gender minority students in Brazil. It noted in the methods section: “First, students were asked “What was your sex assigned at birth?” with the options of male, female, or other… Students were then asked “What is your gender identity?” Their four response options included (a) male, (b) female, (c) travesti or transexual, or (d) other” [44]. The authors also provided their rationale for why they chose to offer the particular answer categories, discussing the meaning of travesti and transexual in the Brazilian context.

The papers that provided the questions showed that the wording and options offered varied, even if the same question format was used. For example, a study examining the mental health status of secondary school students in Suzhou, China also used the two-step method but phrased the questions differently and offered different answer options to the Brazilian study. Respondents were asked “What was your biological sex assigned at birth (choose from male or female)?” and then, “What do you perceive your gender to be (choose from male, female, neither, or not sure)?” [45]. The authors then explain how based on the answers, students were categorized as either cisgender, transgender, non-binary, or questioning. For one paper that used the two-step approach, sex at birth was part of the eligibility criteria rather than an actual survey question [46].

A few of the papers (n = 5) drew on survey data that asked a combined gender identity and sexual orientation question. Four of these papers focused on South East Asia; three on Thailand and one on Myanmar. The authors generally explained this rationale by discussing the difficulty of delineating sexual and gender identities culturally. For example, a study in Chiang Mai, Thailand offered participants eight options for a composite sexual/gender identity question: heterosexual, gay, *kathoey*, *tom*, *dii*, bisexual, questioning, and other [32]. The authors explain that “the term *kathoey* has been used for at least the last several decades to describe a feminine male person who is sexually attracted to men” and that kathoey is understood by some as a third gender in Thailand [32]. They added that the terms *tom* and *dii* could be translated to butch and femme respectively, categories that primarily reflect sexual orientation but also gender expression. In total, ten studies discussed using locally specific gender identity categories with all of these studies either from South East Asia or Brazil. The one study from outside of South East Asia that used a composite question and did not offer locally specific terms was the study on self-harm amongst LGBT amongst adolescents in Ghana in which respondents were offered the options of heterosexual, lesbian, gay, bisexual, or transgender [42].

Four studies drew on survey data that included questions on gender expression. Two of these studies focused exclusively on transgender populations while the other two drew on the same survey data of students in Thailand. In this survey, male respondents were asked to rate themselves in terms of “manliness” and female respondents in terms of “womanliness” compared to their peers [47, 48]. The two papers also represented a trend amongst papers drawing on the same data both to present somewhat different details about the survey design as well as to use the data differently. In one study, the authors discussed and drew on only the gender conformity question [47] while the other incorporated both the gender conformity question and the gender identity question [48].

Crafting appropriate questions around gender identity and expression benefits from knowledge of the cultural context [11]. While most papers did not discuss this point explicitly, around a third (n = 12) described working with local sexual and gender minority experts and populations at some stage of the research design. The majority of these did so through partnering with organizations and experts that serve sexual and gender minority populations. For example, one study on the mental and sexual health of transgender youth in Thailand worked with several transgender associations in Bangkok throughout the research design process. The researchers first met with the associations to discuss the study’s aims, methodology, and ethical considerations; then, they conducted a pre-test of the survey with 30 transgender young people before distributing the revised questionnaires through the associations to a final sample of 200 transgender young people [49].

### Thematic areas

The studies centered around a few key thematic areas. The two most common were sexual health and bullying/violence—each of these themes comprised about thirty percent of the articles (n = 9 for sexual health and n = 8 for bullying/violence)—followed by mental health (n = 8) and substance abuse (n = 4). While some of the articles explored solely one of these themes, others studied several at once, exploring the connections between them.

Often written by public health scholars, the sexual health studies primarily examined sexual health behavior, including questions around number of partners, condom use, and sexually transmitted infections (STI) testing. Additionally, HIV prevention and testing emerged as a central concern: only one of the articles on sexual health did not reference HIV in the title. Compared to the other themes, articles focused on sexual health were more likely to use survey data collected as part of an intervention. For example, one article drew on cross-sectional survey data collected from participants of a UNICEF-funded voucher initiative that aimed to facilitate access to sexual and reproductive health services amongst young MSM and transgender people in Dhaka, Bangladesh [50]. This study is also illustrative of the fact that the sexual health articles tended to study the experience of transgender populations alongside MSM. Only one of the articles on sexual health focused entirely on transgender or gender minority populations. Some of the studies point out how this more secondary focus on gender minority populations might mean that the surveys are less well-suited in assessing their specific needs and experiences [51]. Indeed, the authors of the Bangladesh study noted that the intervention had much greater success finding young MSM than young transgender people, speculating that this might be a result of the study not having engaged leaders or teachers (*guru*) in the *hijra* community to which much of the transgender population in Bangladesh belongs [50].

Most (n = 7) of the bullying/violence articles focused on schools, shedding light on the treatment of school peers and teachers towards gender minority students, while three articles also looked at gender minority youth’s experience in their families. One article took a broader approach, asking about classmates and family, but also about respondents’ experiences in the workplace, healthcare system, and the general public [52]. There was some overlap between the articles focused on bullying/violence and those on mental health with studies in both groups examining linkages between victimization and different psychological difficulties, including low self-esteem, depression, and suicide ideation. Compared to those focused on sexual health, articles focused on bullying/violence and mental health were more likely to include cisgender and heterosexual populations alongside gender and sexual minorities, facilitating comparisons in experience between groups of youth. For example, one study on the mental health of secondary school students in China found that transgender girls were significantly more likely to be bullied and have had suicidal thoughts compared to cisgender girls [45].

A handful of studies focused on alcohol and drug use (n = 4), often showing how gender minority youth’s victimization contributes to these behaviors. For instance, an online survey of Brazilian transgender youth found that discrimination and instability at home were the main drivers of substance use [40]. Similarly, a study of students in Thailand found that social victimization because of sexual orientation or gender identity was associated with a higher likelihood of drug use [53].

Another small portion of studies (n = 4) did not fit within these main themes. Two of these articles focused on questions of identity, one in relation to LGBTQ identity and religiosity in the Philippines [54] and one outlined broad trends in gender identity amongst youth in Chiang Mai, Thailand as well as the meanings attached to various gender identity categories [32]. One study focused on testing the validity of psychological measures related to gender identity in China [55]. Lastly, one study in Brazil examined the academic engagement of gender minority students compared to their cisgender peers [44].

## Discussion

Survey data can provide valuable insights on the experience of gender minority adolescents and youth, allowing for the design of policies that support this population. But, in order for survey data to be used in this way, surveys need to include sex and gender-related measures that allow respondents who identify outside of a cisgender binary to be identifiable in the data. Against this backdrop, we provide an overview of the existing survey research that engages gender minority adolescents and youth in LMICs. The review covers the geographic distribution of this research as well as its key themes, but mostly focuses on its methodological aspects, including how gender identity is assessed in these surveys. This type of analysis is particularly salient at the moment as some studies have found that gender minority youth have faced additional challenges during the COVID-19 pandemic, including in the realm of mental health [56].

Our scoping review reveals a small but quickly growing field that focuses on a few key themes, notably sexual health, mental health, and bullying and violence. While geographically disparate, most of the survey research reviewed came from middle-income countries, suggesting that our knowledge about gender minority youth in low-income contexts is particularly limited. In addition, some regions were not represented in the reviewed articles, including North Africa and the Middle East. One limitation of this review is the focus on articles published in English, which might have impacted the geographic spread of the articles. It is also possible that the review might have missed studies in English that did not use our English-language search terms for gender minorities, but instead exclusively used a term in another language.

In terms of methods, the majority of the studies, including those based on survey data, relied on some form of non-random network-based sampling. As a result, much of this research is limited in making claims about how the experience of transgender and non-binary youth compares to cisgender youth. This suggests that studies that take a broader population approach through probability sampling could valuably contribute to this emerging field. Large existing surveys, such as the Demographic and Health Surveys (DHS), with their strong research infrastructure could play an important role here. One potential first step would be to include a broader gender identity question with a sub-sample of respondents and to explore its reception and accuracy.

At the same time, it is clear that conducting this type of research is complex, culturally contingent as well as potentially risky for researchers and research participants [27]. However, this review included studies in countries where same-sex relations are criminalized, which has repercussions for the wellbeing of gender minority populations. These studies thus show that survey research that captures the experience of gender minority youth is possible in these contexts. One way that study authors aimed to take into account the cultural specificity of dynamics around sexuality and gender is to partner with sexual and gender minority experts and community members at the study site, echoing public health scholars call for a “participatory population perspective” on transgender health [57]. Some of the studies simultaneously worked with youth populations in the research design phase. These types of collaboration may be particularly helpful in identifying appropriate wording of gender identity questions, including the categories offered.

With this small body of survey research on gender minority youth, it is too early to make a blanket recommendation to introduce more nuanced questions on gender identity on all population surveys of youth in LMICs. Further research that tests the resonance and efficacy of different gender identity questions in a wider range of contexts would be helpful in this regard. Some innovative research has already begun, but requires sustained funding [11, 20]. We also suggest that researchers continue to outline in articles how gender identity was asked about in their survey data. These additional details could include providing the survey questions on gender identity and their accompanying answer options, as well as outlining any methodological challenges that emerged in this process of designing and administering the questions. Given the relatively recent emergence of such questions as well as their cultural contingency, such information could provide other researchers interested in including more nuanced gender identity questions with ideas of how to do so.

## Supporting information

S1 Fig PRISMA diagram. (TIF)

S1 Table Characteristics of included studies. (XLSX)
