# Supplementary figures and images for: A scoping review of survey research with gender minority adolescents and youth in low and middle-income countries
Source: PLoS One. 2023 Jan 10;18(1):e0279359. doi: 10.1371/journal.pone.0279359 (PMC9831317; doi:10.1371/journal.pone.0279359)

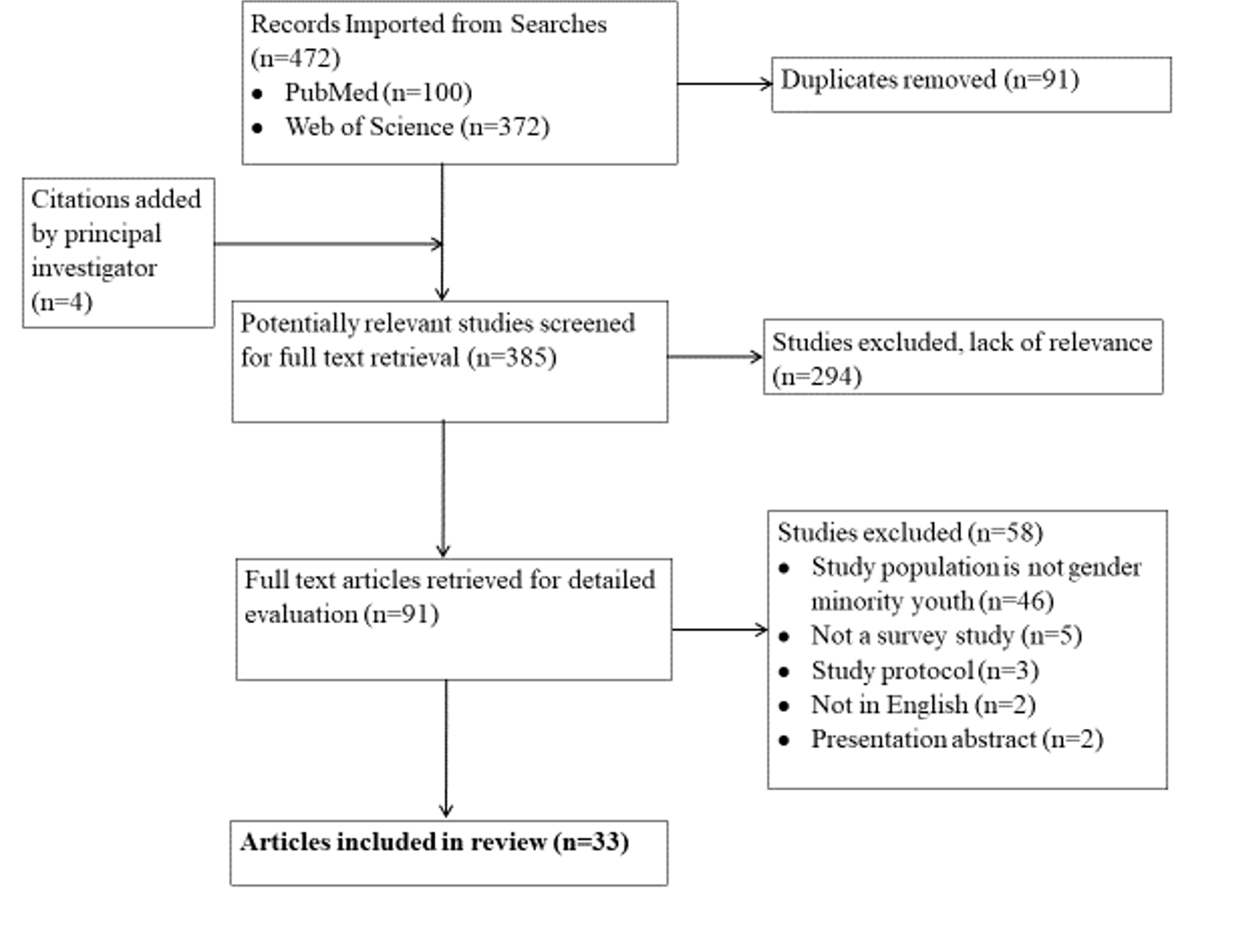

Supplement: S1 Fig — (TIF) [file pone.0279359.s001.tif]
